# How small is the smallest? New record and remeasuring of *Scydosella musawasensis* Hall, 1999 (Coleoptera, Ptiliidae), the smallest known free-living insect

**DOI:** 10.3897/zookeys.526.6531

**Published:** 2015-10-08

**Authors:** Alexey A. Polilov

**Affiliations:** 1 Department of Entomology, Faculty of Biology, Lomonosov Moscow State University, Moscow 119234, Russia

**Keywords:** Smallest beetle, body size, SEM, Colombia

## Abstract

The smallest known beetle *Scydosella musawasensis* Hall is recorded for the second time. Precise measurements of its body size are given, and it is shown that the smallest examined representative of this species has a length of 325 µm.

## Introduction

The smallest insects have recently attracted considerable attention as models for studying animal miniaturization, since they are among the smallest metazoans and since many morphological features unique to them and resulting from their extremely small size have been described (Polilov 2015). The size of the smallest known parasitoid insect, male *Dicopomorpha eschmepterigis* (139 µm) is known rather precisely, and has been determined by using modern equipment (Mockford 1997; Huber and Noyes 2013). On the other hand, it is not quite clear which free-living insect is the smallest. It is stated in a great number of scientific and popular publications that the body length of the smallest beetles is 0.25 mm, but this statement is incorrect, although it has a long history. LeConte (1863) described *Ptilium fungi*, specifying its length as ‘scarcely more than 1-100 of an inch,’ i.e., 254 µm. Motschoulsky (1868) almost simultaneously described *Nanosella fungi* from Georgia, North America, specifying its length 1/10 l., i.e., 212 µm (1 line = 2.116 mm). Sörensson (1997), who re-examined the type material, indicated that the length given in earlier studies resulted from error of measurement and undescribed *nanosellines* remained the smallest, having a body length of about 0.3 mm (Dybas 1990). Therefore, it was still unclear which known beetle was the smallest. Hall (1999) described several new genera and species; as a result, *Scydosella musawasensis* Hall, 1999, which has a body length of 0.30 mm, became the smallest described beetle. This species was known up to date only from several specimens of the type series collected by B. Malkin in Nicaragua. *Scydosella musawasensis* was measured only from cleared specimens embedded in preparations for microscopy studies, which makes it difficult to measure length precisely.

## Methods

Adults of *Scydosella musawasensis* Hall, 1999 were collected in Chicaque National Park, Colombia, 10 km west of Bogotá, on 8 February 2015 (coordinates 4.619, -74.312), 2200 m above sea level, on the fungus *Steccherinum* sp. (Meruliaceae), 85 specimens. The material was fixed in FAA (formaldehyde—alcohol—acetic acid) and preserved in 70% ethanol. It was subsequently examined under a Jeol JSM-6380 scanning electron microscope (SEM) after drying of the specimens at the critical point (Hitachi HCP-2) and sputter coating with gold (Giko JSM-6380). The measurements were made using the program Meazure (C Thing Software) from digital micrographs obtained under SEM.

## Results and discussion

**Figure 1.**
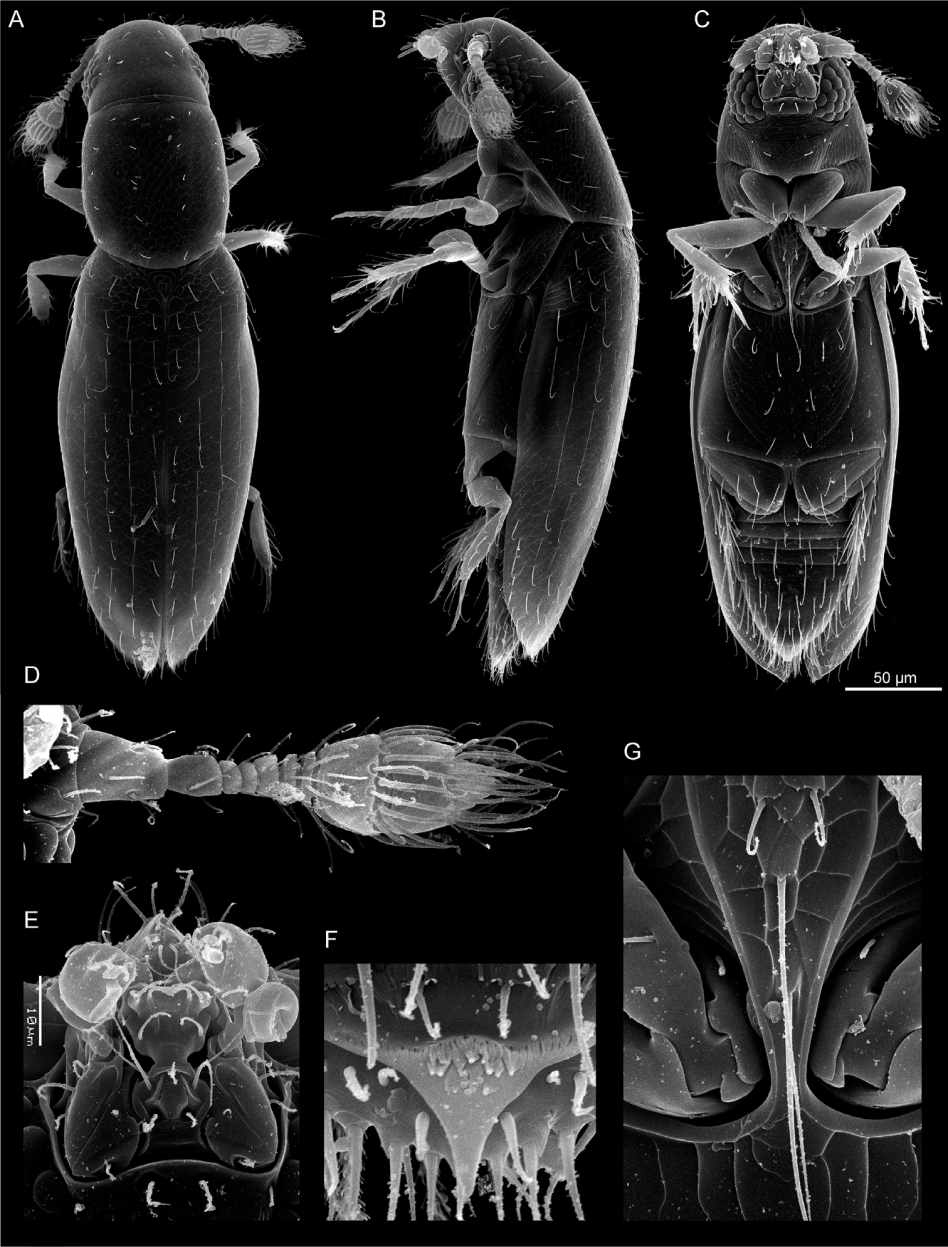
Habitus and diagnostic characters of *Scydosella musawasensis*, SEM: **A** dorsal view **B** lateral view **C** ventral view **D** antenna **C** mouthparts **F** pygidial tooth **G** mesosternal process.

Measuring of ten specimens of *Scydosella musawasensis* has shown that the smallest of them has a length of 325 µm, the largest has a length of 352 µm, and the average length is 338 µm (Fig. 1). The body width (maximum width of both elytra at rest) is 98 to 104 µm (М = 99 µm, n = 10). Thus, the smallest beetle and the smallest known free-living insect has a body length of 325 µm.

The record of *Scydosella musawasensis* in Colombia considerably broadens the known range of this genus and species, known previously only from one site in Nicaragua (Hall 1999), where the type series was collected: Musawas, Waspuc River, Nicaragua, 14 October 1955. This record also broadens the known range of fungi colonized by *Scydosella musawasensis*, which was known previously only from *Rigidoporus lineatus* (Meripilaceae, given as *Polyporus zonalis* in the original description) (Hall 1999); I have collected it on *Steccherinum* sp. (Meruliaceae).

This genus and the only described species it includes differ from the other Nanosellini in the following combination of characters. Body elongate-oval (Fig. 1A–C), yellowish-brown, surface generally glabrous, punctation sparse. Antennae 10-segmented (Fig. 1D). Mentum setal formula 2+2+1 (Fig. 1E). Pronotum widest at middle. Procoxal pockets absent, prothoracic glands absent. Mesosternal process evenly narrowing anteriad, with obtuse apex, not extending onto metasternum (Fig. 1G). Mesosternal lines ending near process; metasternal lines complete. Elytral venter with stridulatory file. Femoral line ending in 2 setae. Pygidial tooth acute (Fig. 1F). Spermatheca rounded, as described earlier (Hall 1999: p. 123, no. 147).
